# Optimization of fermentation conditions for production of l‐arabinose isomerase of *Lactobacillus plantarum* WU14

**DOI:** 10.1002/fsn3.1989

**Published:** 2020-11-24

**Authors:** Zhijun Sun, Tingting Miao, Aiguo Yin, Hulin Qiu, Yunyi Xiao, Ying Li, Jinping Hai, Bo Xu

**Affiliations:** ^1^ College of Biological and Food Engineering Guangdong University of Petrochemical Technology Maoming China

**Keywords:** d‐tagatose, fermentation kinetics, *Lactobacillus plantarum* WU14, l‐arabinose isomerase

## Abstract

As a substitute sweetener for sucrose, d‐tagatose is widely used in products, such as health drinks, yogurt, fruit juices, baked goods, confectionery, and pharmaceutical preparations. In the fermentation process of l‐AI produced by Lactobacillus plantarum, d‐tagatose is produced through biotransformation and this study was based on the fermentation process of *Lactobacillus plantarum* WU14 producing l‐AI to further research the biotransformation and separation process of d‐tagatose. The kinetics of cell growth, substrate consumption, and l‐arabinose isomerase formation were established by nonlinear fitting, and the fitting degrees were 0.996, 0.994, and 0.991, respectively, which could better reflect the change rule of d‐tagatose biotransformation in the fermentation process of *L. plantarum* WU14. The separation process of d‐tagatose was identified by decolorization, protein removal, desalination, and freeze drying, initially. Finally, the volume ratio of whole cell catalysts, d‐galactose, and borate was 5:1:2 at 60°C, pH 7.17 through borate complexation; then, after 24 hr of conversion, the yield of d‐tagatose was 58 g/L.

## Graphical abstract

*Lactobacillus plantarum* WU14 was isolated from sauerkraut, and it has high ability to transform d‐tagatose by l‐arabinose isomerase. On the base of the fermentation medium optimization of l‐AI produced by *L. plantarum* WU14, a mathematical model was established, and d‐tagatose was separated from the fermentation broth. The Logistic equation and the Boltzmann model were used to nonlinearly fit the experimental data of *L. plantarum* WU14 biomass and l‐AI activity. It was found that when the conversion temperature was 60°C, the reaction pH was 7.17, the volume ratio of the crude enzyme solution to the d‐galactose was 5:1, and the volume ratio of borate to d‐galactose was 2:1. After 24 hr of conversion, the conversion of d‐tagatose was increased 12%.

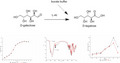

## 1 INTRODUCTION

d‐Tagatose is a new functional sweetener, it is the first rare no sugar alcohol that has been industrially manufactured in nature (Marion et al., 2018; Xu et al., 2018), which has the functions of lowering blood sugar (Donner et al., 2010), improving intestinal flora, anticaries, promoting blood health, inhibiting cocaine and other substances on liver toxicity, reducing energy, and reducing obesity (Lu & Levin, 2002; Normen et al., 2001). Currently, d‐tagatose can be used as a substitute for sucrose and widely used in food industry, such as yogurt (Torrico et al., 2019), fruit juices, baked goods, and candies (Levin, 2002). In addition, it can also be used in beauty and pharmaceutical industries (Jayamuthunagai et al., 2016).

l‐arabinose isomerase (l‐AI) is an effective enzyme for the industrial conversion of d‐galactose to d‐tagatose (Roh et al., 2000). Since 1993, Cheetham and Wootton (1993) fermented d‐galactose to d‐tagatose by microbial l‐AI. Nowadays, the d‐tagatose biotransformation research of l‐AI has become a hot spot and it has been reported that strains which could produce d‐tagatose by l‐AI, include *Escherichia coli* (Babu & Manjasetty, 2006), *Bacillus stearothermophilus* US100 (Rhimi et al., 2009), *Geobacillus thermodenitrificans* (Oh et al., 2006), *Lactobacillus plantarum*, *Thermotoga neapolitana* (Kim et al., 2002), and so on. Several researches of l‐AI have been done worldwide. l‐AI from thermophilic bacteria has been deeply analyzed by both Hung et al. (2014) and White et al. (2017) At the same time, Prabhu et al. (2010) used homology modeling for site‐directed mutation of l‐AI. In order to improve the catalytic efficiency of d‐galactose, boric acid was added into the l‐AI enzyme of Anoxybacillus flavithermus by Li et al. (2011); meanwhile, Xu et al. (2012) increased the conversion of d‐tagatose to 60% under boric acid conditions. Depending on immobilized enzyme technique, yield of d‐tagatose has been promoted to 22.3% and 43.9% by Patel et al. (2017) and Jebors et al. (2011) and Jayamuthunagai et al. (2017) used whey permeate to immobilize Lactobacillus plantarum cells to rise the conversion of d‐tagatose to 38% and 33%. While Guo et al., (2018) increases the conversion of l‐AI to 79.7% by exploiting the anchoring protein Cot G with a peptide linker to boost the conversion of l‐AI to 79.7%, Hao et al. (2019) enhances the conversion of L‐arabinose by coexpression system with l‐AI and d‐RI.

Because of the large population base and rapid aging trend in the world, the potential of the diabetes drug market has further increased. Therefore, the development of domestic products is extremely urgent and the market prospect is very broad. Based on the optimization of the fermentation medium and culture conditions, the enzyme activity of l‐AI from *L. plantarum* WU14 with high d‐tagatose yield was reached to 13.75 U/ml (Chang et al., 2016), which was isolated from sauerkraut. In the early study, the fermentation process of *L. plantarum* WU14 producing l‐AI was optimized using single‐factor and response surface experiments, the biomass of *l. plantarum* WU14 was 2.52, and the enzyme activity of l‐AI was 42.23 U/ml, respectively (Zhijun et al., 2019). This study focused on biotransformation and separation process of d‐tagatose, and it would lay the foundation for the industrial production of d‐tagatose bioconversion in the future.

## 2 MATERIALS AND METHODS

### 2.1 Preparation of whole cell catalysts

*Lactobacillus plantarum* WU14 was inoculated into MRS medium at 1%, activated at 37°C, and inoculated into the fermentation medium (the fermentation medium was composed of glucose 4.35 g, l‐arabinose 2.71 g, yeast extract 17.6 g, soy peptone 17.6 g, anhydrous sodium acetate 10 g, MgSO_4_.7H_2_O 0.4 g, MnSO_4_.2H_2_O 0.05 g, K_2_HPO_4_ 0.4 g, pH 6.24) at 2%, then was fermented at 37°C for 24 hr. The fermentation broth was centrifuged at 4°C for 10 min (9,000 r/min), the supernatant was discarded, the cells were washed twice with phosphate buffer (0.2 M, pH 7.17) and were resuspended in phosphate buffer finally, the final volume was 1/10 of the fermentation broth, and the obtained resuspended bacterial solution was a whole cell catalysts solution and was stored at 4°C.

d‐tagatose content: according to the Cysteine–carbazole method, mix 0.5 ml of whole cell catalysts with 0.5 ml of 0.8 M galactose substrate solution (containing 10 mM MnCl_2_), after incubate in the solution at 60°C 24 hr, then terminate the reaction, and determine the d‐tagatose content according to the standard curve.

### 2.2 The fermentation kinetics of l‐arabinose isomerase from *Lactobacillus plantarum* WU14

The seed solution was inoculated into the fermentation medium at 2% and fermented at 28°C. The biomass, reducing sugar content, pH, and l‐AI activity were measured every 2 hr. The Origin 9.1 software would be used together with the Logistic equation and Boltzmann model to nonlinearly fit the experimental data, and the growth kinetics, substrate consumption kinetics, and l‐AI generation kinetics model were established.

### 2.3 Separation and purification of d‐tagatose

The fermentation broth was a mixture of d‐galactose and d‐tagatose. First, the bacteria removed by centrifugation, and activated carbon, ethanol, and trichloroacetic acid (TCA) were added to the fermentation broth to determine the best decolorization and deproteinization process, then use anion and cation exchange resin, Ca^2+^ resin, freeze drying, etc. to d‐tagatose, and finally perform spectral analysis.

### 2.4 Borate catalyzes production of d‐tagatose

A borate buffer solution was added to the d‐tagatose conversion system; then, the conversion temperature, the reaction pH, the amount of the whole cell catalysts, and the amount of the borate buffer solution were determined.

### 2.5 Method for measuring each index

1. l‐AI activity: Cysteine–carbazole method, d‐tagatose standard curve equation: *y* = 0.0239*x* + 0.0244 (*R* ^2^ = 0.9987).
2. Reducing sugar content: DNS method, standard curve equation: *y* = 1.1719*x* − 0.0194 (*R* ^2^ = 0.9964).
3. Protein content: Coomassie Brilliant Blue G‐250 method, standard curve equation: *y* = 8.0597*x* + 0.0502 (*R* ^2^ = 0.9856).

## 3 RESULTS AND ANALYSIS

### 3.1 Analysis on l‐AI fermentation kinetics model

#### 3.1.1 The changes in various parameters during fermentation

##### Changes of bacterial cell biomass during fermentation

**Figure 1:**
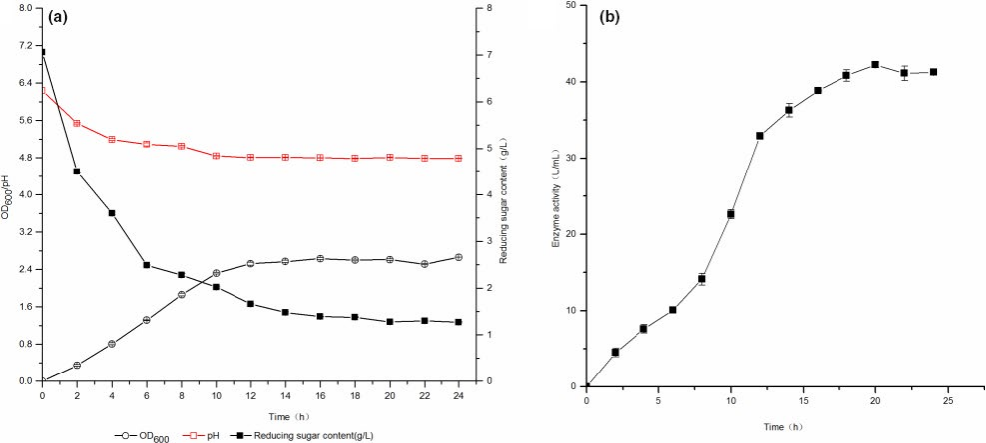
The changes in parameters during fermentation. (a) The changes in OD_600_, pH, and reducing sugar content; (b) The changes inl‐AI enzyme activity

The change of cell biomass during fermentation is shown in Figure 1a. From the growth curve of *L. plantarum* WU14 in the figure, it could be seen that *L. plantarum* WU14 grows in the “S” type, the cells had obvious logarithmic and stable growth period, and the adaptation period was not obvious. The biomass of *L. plantarum* WU14 reached 0.346 after 2 hr fermentation and entered the logarithmic growth stage. With the increase of fermentation time, the biomass of *L. plantarum* WU14 increased continuously; after 12 hr, it reached OD_600_ of 2.52 and entered the stable growth stage. During the stable period, the biomass of *L. plantarum* WU14 remained unchanged basically.

##### The changes in pH during fermentation

From the pH curve of Figure 1a, the pH showed a decline trend. When fermented for 12 hr, and the pH value decreased from 6.24 to 4.8, after 12 hr, the growth of *L. plantarum* WU14 entered the stable stage, and the pH value decreased slowly. In addition, although the pH of the fermentation broth was decreasing continuously, the final was more than 4.5, because l‐arabinose induced *L. plantarum* WU14 to produce l‐AI in the medium, *L. plantarum* WU14 was not mainly acid degradation mechanism.

##### The changes in reducing sugar during fermentation

The role of the carbon source in the medium is to provide the carbon skeleton of the cell, to provide energy for the life of the cell, and to provide a carbon skeleton for the synthesis of the product. It could be seen from the reducing sugar content curve of Figure 1a, and the reducing sugar content showed a gradual decline trend. In the early stage of fermentation, reducing sugars were used for cell proliferation, and the concentration of reducing sugar decreased rapidly. When the cells reached a stable period, reducing sugars only provided energy for their life activities, and the content basically stabilizes.

##### The changes in l‐AI activity during fermentation

From the l‐AI activity curve of Figure 1b, it could be seen that the activity of l‐AI enzymes increased first and then decreases. There was a correlation between the biomass and the activity of enzymes in the initial fermentation (0–12 hr). After 12 hr, the cell reached a stable stage, and the biomass did not increase; however, the enzyme activity continued increasing. The reason was the expression of l‐AI was affected by the regulation of the arabinose operon (Sheppard & Englesberg, 1967). The *araA*, *araB,* and *araD* genes in the arabinose operon encode three enzymes were required for arabinose metabolism: arabinose isomerase, ribokinase, and ribone‐5‐phosphate isomerase, respectively. The expression of the three genes was regulated by *ara*C in the arabinose operon. In the initial stage, the concentration of glucose was high in the medium, which led to the low concentration of cAMP, *araC* inhibited the transcription of *araA*, *araB,* and *araD*. CAP‐cAMP complex could not bind to the CAP binding site in the operon, so *araA* could not express normally, and the activity of l‐AI was relatively low. Due to the rapid growth of logarithmic cell and the consumption of large amounts of glucose, the concentration of glucose reached a relatively low level after 12 hr fermentation, and l‐arabinose was at a high concentration level of 2.71 g/L; then, the CAP‐cAMP complex could bind to the CAP binding site in the operon, which resulted the normal expression of *araA*. The activity of l‐AI continued to increase and reached the maximum activity of 42.23 U/ml at 20 hr, and the yield of d‐tagatose was 2.5338 mg/(ml hr), 49.68g/(L·d). The arabinose was already at a lower level after 20 hr, and *araC* became a transcriptional repressor of *araA*, *araB,* and *araD*, which prevented the normal expression of *araA*, and the activity of l‐AI declined. It could also show that the production of l‐AI and the growth of cells were nongrowth‐coupled.

#### 3.1.2 Establishment of fermentation kinetics model

##### Establishment of cell growth model

The growth of *L. plantarum* WU14 was a “S” type, so the Logistic model and the Boltzmann model (Jia et al., 2012) were used to dynamically fit the growth curve.

By using the Origin 9.1 software, the kinetic parameters *μ*_m_ and *X*_max_ were 0.28687 and 37.77377, respectively, by Logistic model, and *R* ^2^ = 0.9763. The kinetic parameters *A*_1_ = −0.19407, *A*_2_ = 2.62428, *x*_0_ = 5.52242, *d*_x_ = 2.29839 by Boltzmann model, and *R* ^2^ = 0.99637. The fitting with the Logistic model was worse than the Boltzmann model, because *L. plantarum* WU14 had no obvious adaptation period in the fermentation process and entered the phase of logarithmic growth shortly after inoculated, so the growth curve did not belong to the typical “S” type.

The Boltzmann model was selected to describe the growth of *L. plantarum* WU14 in fermentation process, and the maximum biomass of bacteria was 2.62428.

The Boltzmann equation was *y* = (*A*_1_ − *A*_2_)/(1 + *e* ^(x‐x0)/dx^) + *A*_2_.

*A*_1_: the initial concentration of biomass;

*A*_2_: the final concentration of biomass;

*x*_0_, dx: the equation coefficients.

The growth kinetic model of *L.plantarum* WU14 was: *y* = 2.62428 − [2.81835/(1 + e(*x* − 5.52242)/2.29839].

**Figure 2:**
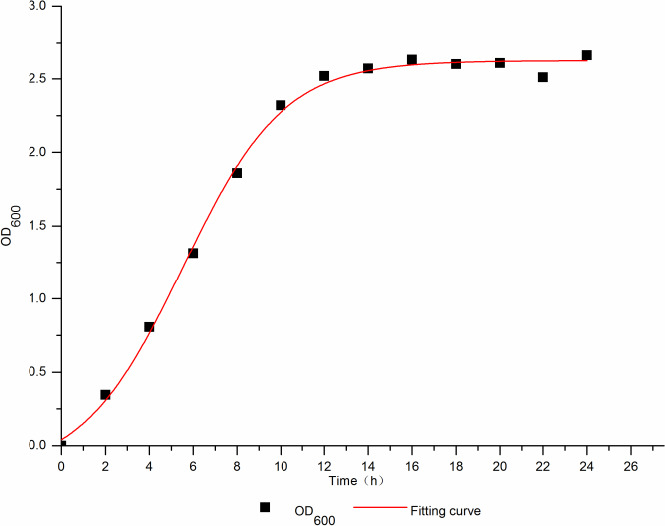
The growth kinetic model of*L. plantarum* WU14

The fitting curve is shown in Figure 2, the growth fitting value of *L. plantarum* WU14 is basically consistent with the experimental value, and this model could better reflect the growth of the bacteria in the batch fermentation process of *L. plantarum* WU14.

##### **Establishment of** l **‐AI generation dynamics model**

Used the Leudeking–piret equation (Luedking & Piret, 1959), the kinetic parameters *μ*_m_, *X*_max_, *α*, and *β* were 0.28687, 37.77377, 5.92606, and 1.23832, respectively, and *R* ^2^ = 0.94441; the Boltzmann model was used for dynamic fitting, and the values of kinetic parameters *A*_1_, *A*_2_, *x*_0,_ and dx were 1.66015, 41.94723, 9.53752, and 2.46001, respectively, with the fitting degree *R* ^2^ = 0.99116. By combining the two fitting degrees, the formation of l‐AI was well fitted with the Boltzmann model, and the maximum l‐AI enzyme activity was 41.94723 U/ml. Therefore, the Boltzmann model was chosen to fit l‐AI.

**Figure 3:**
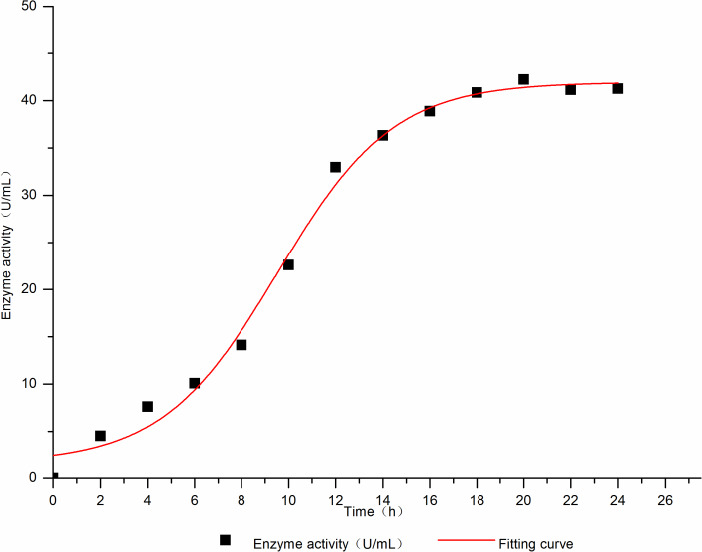
Thel‐AI enzyme activity mechanics fitting

The kinetic equation of l‐AI could be obtained as follows: *y* = 41.94732 + [(−40.28708)/(1 + e^(x−9.53752)/2.46001^]. The fitting curve is shown in Figure 3.

##### Establishment of substrate consumption kinetics model

The consumption of substrate during fermentation has three aspects (Jinghong, 2007): (a) consumption of bacterial growth; (b) maintenance of bacterial basic life activities; and (c) consumption of synthetic products.

The Boltzmann model was used for dynamic fitting, and the values of kinetic parameters *A*_1_, *A*_2_, *x*_0,_ and dx were 11,297.04, 1.30758, −31.55851, and 4.15091, respectively, with the fitting degree *R* ^2^ = 0.99171; and by using the Logistic model, the kinetic parameters *A*_1_, *A*_2_, *x*_0,_ and p were 7.04942, 0.6189, 3.06749, and 1.14414, respectively, *R* ^2^ = 0.99443. Therefore, the substrate consumption was well fitted to the Logistic model, which could better reflect the change of reducing sugar content during fermentation.

The Logistic model equation was *y* = (*A*_1_ − *A*_2_)/(1 + (*x*/*x*_0_)^p^) + *A*_2_.

*A*_1_: the initial sugar content;

*A*_2_: the final sugar content;

*x*_0_, p: the equation coefficients.

**Figure 4:**
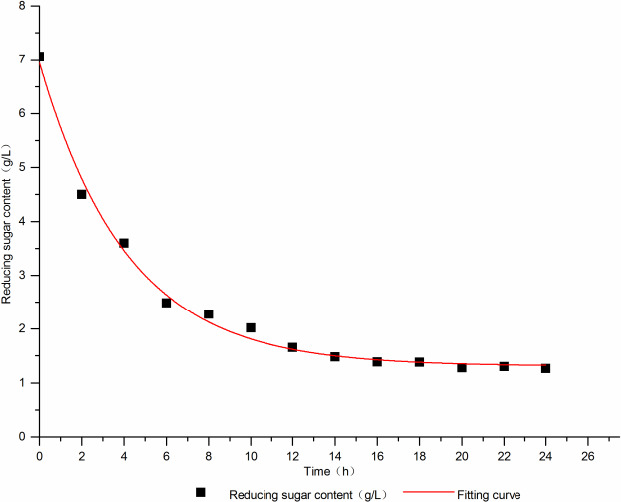
The matrix consumption dynamics fit

The kinetic equation of substrate consumption was:

*y* = 0.6189 + (6.43052)/[1 + (*x*/3.06749)^1.14141^]. The fitting curve is shown in Figure 4.

### 3.2 Study of the separation and purification of d‐tagatose

#### 3.2.1 The pretreatment of the fermentation broth

The fermentation broth was centrifuged for 30 min (4°C, 9,000 rpm) take the supernatant, which was a mixture of d‐tagatose and d‐galactose, and stored at 4°C.

#### 3.2.2 The decolorization process of the fermentation broth

##### The effect of the amount of activated carbon on the decolorization

**Figure 5:**
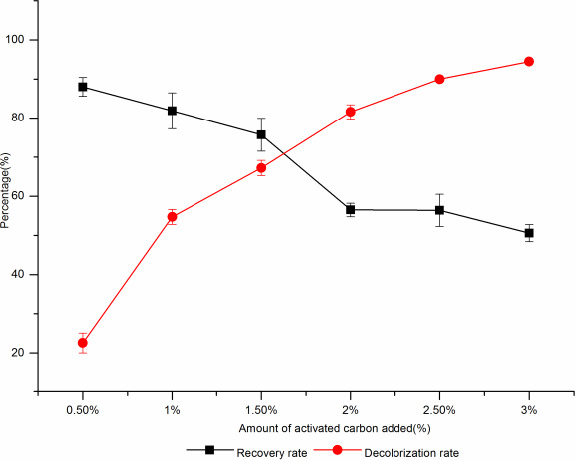
The effect of activated carbon additive on decolorization rate and sugar recovery rate

Activated carbon decolorization is a commonly decolorization method, because the impurities can be removed by the adsorption of activated carbon. The larger surface area of activated carbon, the stronger adsorption capacity would be got. The decolorization process of activated carbon powder is affected by the decolorization temperature, decolorization time, the amount of activated carbon added, and the pH of the solution. The appropriate amount of activated carbon powder was soaked in 0.4 M HCl solution in room temperature for 24 hr, washed with water to pH neutral, and filtered and dried at 50°C for use (Xinyin, 2014). Different amounts of activated carbon were added to the fermentation broth and kept for 30 min; the decolorization rate of the fermentation broth increased with the increase of activated carbon; however, the recovery rate of d‐tagatose decreased (Figure 5). When the addition amount of activated carbon was 1.5%, the decolorization rate was about 67.21% and the recovery rate of d‐tagatose was 75.7%, and when the additive amount reached 2%, the decolorization rate increased by 14.37% and the recovery of d‐tagatose decreased by 19.2%. Therefore, the optimal additive amount of activated carbon was 1.5%.

##### The effect of decolorization time on decolorization

**Figure 6:**
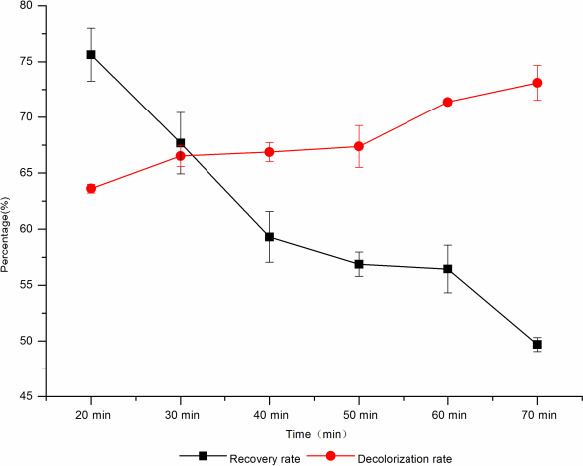
The effect of decolorization time on decolorization rate and sugar recovery rate

The decolorization time has a great influence on the decolorization rate and sugar recovery rate. As the decolorization time increased, the decolorization effect was better. When the decolorization time was 30 min, the decolorization rate was 66.53% and the recovery rate of d‐tagatose was 67.69%, and when the decolorization time was extended to 40 min, the decolorization rate reached 66.87% and the recovery rate of d‐tagatose was 59.31%, decreased by 8.38% (Figure 6). Therefore, the optimal decolorization time was 30 min.

##### The effect of decolorization temperature on decolorization

**Figure 7:**
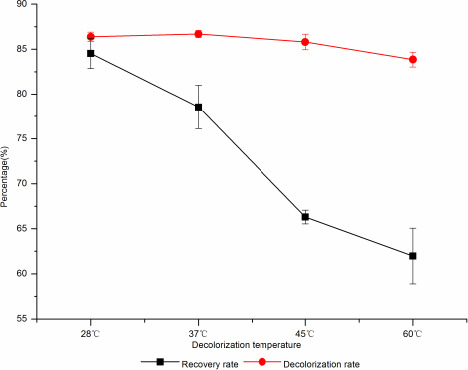
The effect of decolorization temperature on decolorization rate and sugar recovery rate

From Figure 7, the result has shown that the decolorization effect changed little with the increase of decolorization temperature. When the decolorization temperature was 28°C, the decolorization rate was 86.69%, and then gradually decreased, and when the temperature was 60°C, the decolorization rate decreased to 83.85%. The recovery of d‐tagatose decreased greatly with the increase of temperature. When the decolorization temperature was 28°C, the recovery rate was 84.53%, and when the temperature was 60°C, the recovery rate was 61.98%, decreased by 22.55%. Therefore, the optimal decolorization temperature was 28°C.

##### The effect of pH on decolorization

**Figure 8:**
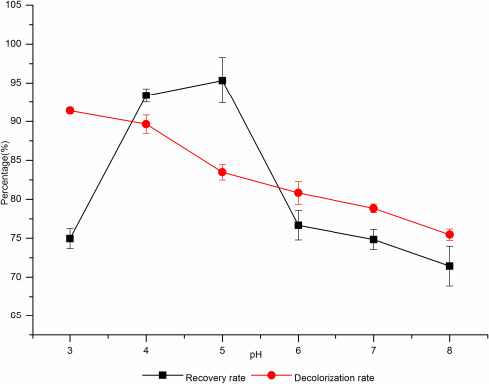
The effect of pH of fermentation broth on decolorization rate and sugar recovery rate

When the pH was 3, the maximum decolorization rate was 91.38%, and then gradually decreased, and when the pH was 8, the decolorization rate dropped to 75.46% (Figure 8). The recovery rate of d‐tagatose increased first and then decreased with the increase of pH. When the pH was 4, the recovery rate was 93.37% and the decolorization rate was 89.64%, and when the pH was 5, the recovery rate was up to 95.33% and the decolorization rate was 83.47%. Therefore, the optimal pH for decolorization was 4.

#### 3.2.3 The study on deproteinization process

##### Low‐temperature ethanol method

**Figure 9:**
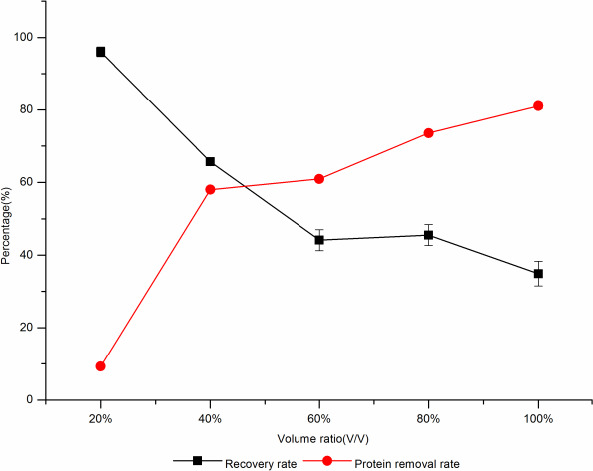
The effect of low‐temperature ethanol on protein removal rate and sugar recovery rate

The protein removal rate increased with the increase of low‐temperature ethanol, and the recovery rate of d‐tagatose gradually decreased. When the additive amount of ethanol was 40%, the protein removal rate was 58%, and the recovery rate was 65.68%; when the additive amount of ethanol increased to 60%, the protein removal rate showed little change and the recovery rate significantly decreased by 21.55% (Figure 9). Therefore, 40% (V/V) was selected the optimal addition.

##### TCA method

**Figure 10:**
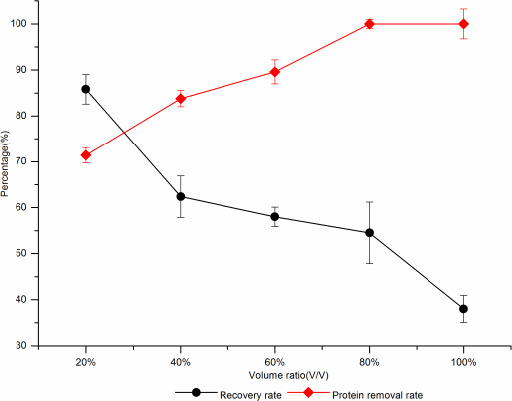
The effect of TCA on protein removal rate and sugar recovery

When the additive amount of TCA was 20%, the protein removal rate was 71.4% the recovery rate was 85.82%, and when the additive amount of TCA increased to 40%, the protein removal rate was 83.8% and the recovery rate decreased by 23.44% (Figure 10). Therefore, 20% (V/V) was selected the optimal addition.

##### Comparison between low‐temperature ethanol method and TCA method

The TCA method was better than the low‐temperature ethanol method in both protein removal and d‐tagatose recovery (Table 1). Therefore, the TCA method was selected the optimal method for protein removal.

**Table 1:** The comparison between low‐temperature ethanol method and TCA deproteinization

	Protein removal rate	Tagatose recovery rate
Ethanol method	58%	65.68%
TCA method	71.4%	85.82%

#### 3.2.4 Anion exchange resin desalting

**Figure 11:**
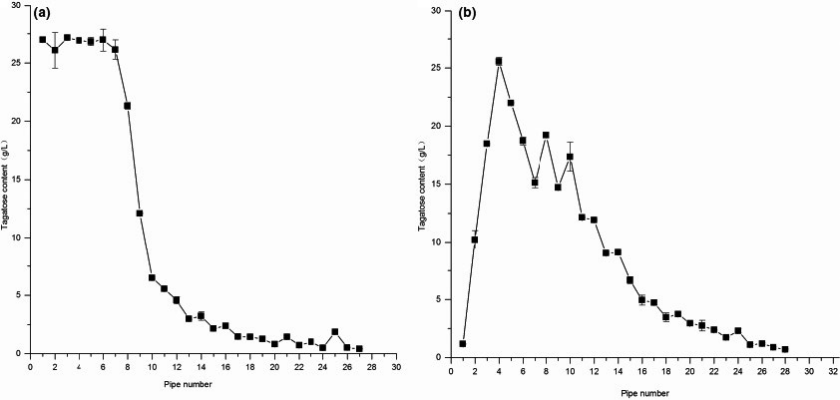
The desalting and decolorization ofd‐tagatose. (a) Cationic resin chromatography; (b) Anion resin chromatography

The treated resin was filled in two 25 mm * 300 mm glass columns (Huang Wenxia et al., 2008; Tie et al., 2000), with column volume of 50 ml, sample volume of 20 ml, and the elution rate of 1 ml/min. The collected results are shown in Figure 11.

Figure 11a shows the eluent obtained by cationic exchange resin chromatography of concentrated sugar solution. The eluent was collected from 16 ml, 1 tube for every 5 ml, and a total of 27 tubes were collected. The concentrations of the sugar liquid collected were the highest of the fifth and sixth tube, and then, the concentration was gradually decreased. At the 20th tube, the concentration of the sugar liquid was 0.

The eluent of 1–20 tubes was collected for anion exchange resin chromatography. The eluent was collected from 10 ml, 1 tube for every 5 ml, and a total of 28 tubes were collected. The collection results are shown in Figure 11b, the highest concentration of eluent was 25.425 mg/ml of 4th tube, and then, the concentration of eluent gradually decreased. At the 28th tube, the concentration of the sugar liquid was 0.7 mg/ml. Finally, the recovery rate of d‐tagatose was 84.22%.

#### 3.2.5 Separation and purification of calcium resin

**Figure 12:**
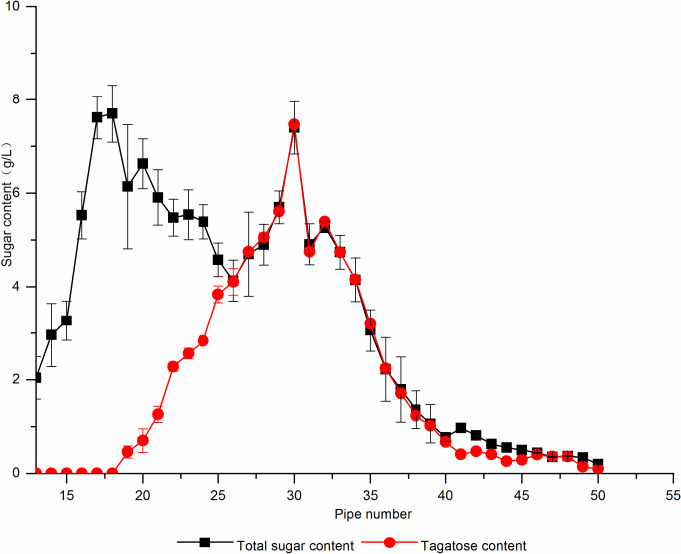
The separation and purification of calcium resin

With the increase of column temperature, d‐galactose and d‐tagatose separation efficiency increases gradually, and the separation effect is best at 70°C (Huang Wenxia et al., 2008). Therefore, after the resin was treated (Zhongyu et al., 2003), the chromatographic column was kept 70°C by circulating water, column volume of 80 ml, sample volume of 1 ml, and the elution rate of 1 ml/min, every 3 ml collection tube, the collected result is shown in Figure 12. At the 13th tube, d‐galactose began to flow out, d‐tagatose flowed out from the 19th tube, and the 19th–25th tube was a mixture of d‐galactose and d‐tagatose. At the 25th–50th tube, there was only d‐tagatose, and at the 30th tube, the elution concentration was the highest, and the recovery rate of d‐tagatose reached 80.8%.

#### 3.2.6 Infrared spectroscopy identification

**Figure 13:**
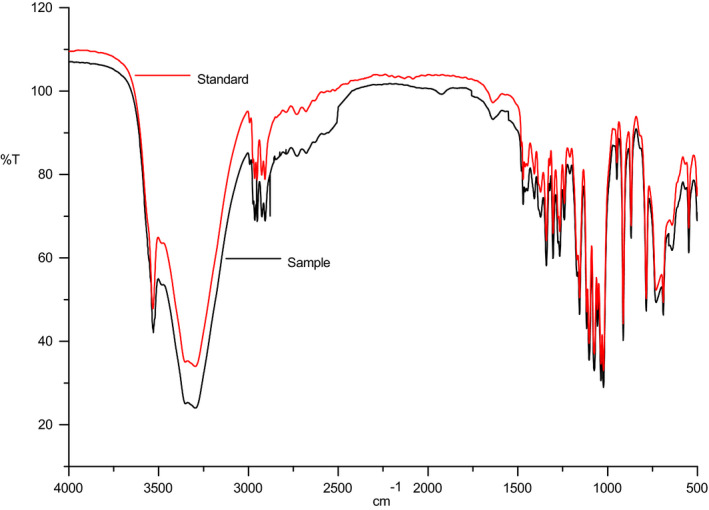
Identification of infrared spectrum

After concentrated and freeze‐dried, the sugar liquid was identified by infrared spectroscopy, and the results are shown in Figure 13. First, the infrared spectra of d‐tagatose samples were consistent with the standard substance; second, the absorption peaks were consistent. Therefore, the purified samples were considered to be d‐tagatose.

### 3.3 Study on the production of d‐tagatose by l‐AI catalyzed by borate

#### 3.3.1 Borate catalyzes the optimal pH of l‐AI

**Figure 14:**
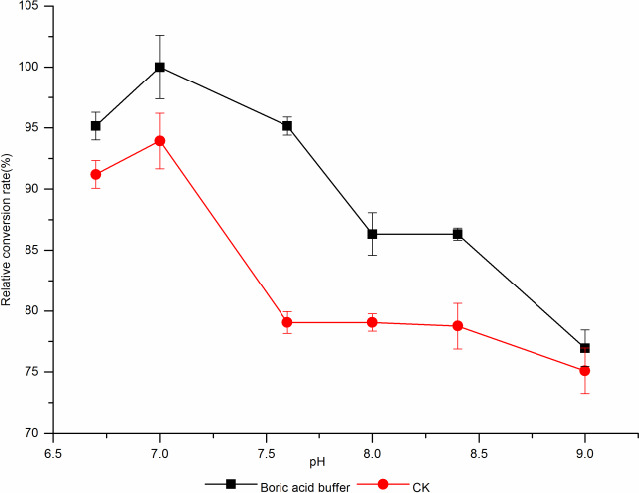
The effect of different pH borate ond‐tagatose

The results are shown in Figure 14: the conversion trend of borate buffer system and phosphate buffer to l‐AI production of d‐tagatose was consistent. The conversion of d‐tagatose under acidic condition was higher than that under alkaline condition, and when the pH was 7.17, the conversion rate of d‐tagatose was the highest. When the pH of the phosphate buffer was increased to 7.6, the conversion of d‐tagatose decreased sharply by 14.88%, it may be that the alkaline buffer inhibited the catalysis of enzyme, with the alkaline buffer increased, the conversion rate of d‐tagatose became lower and lower. Xiaohui (2012) studies that as the basicity of the reaction system increased, the complexing ability of borate to d‐tagatose was better, and the optimal pH of borate for d‐tagatose conversion was 9.0; Lim et al. (2007) studies that the optimal pH for the conversion of d‐tagatose was 8.5. The optimal pH of borate catalyzed *L. plantarum* WU14 l‐AI to d‐tagatose was 7.17. When the pH of borate was increased to 7.6, the d‐tagatose conversion rate decreased by 2% and did not continue to increase, it may be that the transformation of this experiment used the whole cell, not the enzyme protein after ultrasonic broken, *L. plantarum* WU14 was a lactic acid bacteria isolated from sauerkraut, which was more acidic than other strains.

#### 3.3.2 Borate catalyzes the optimal temperature of l **‐AI**

**Figure 15:**
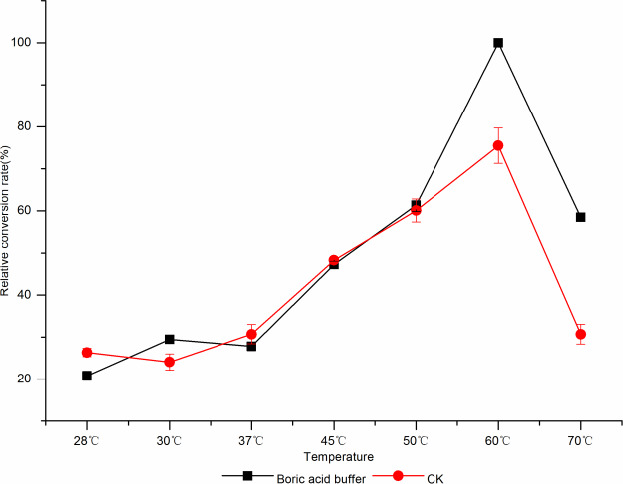
The effect of different temperatures on borate‐catalyzedd‐tagatose

The temperature has a great influence on the enzyme activity and the complexing ability of the conversion products. When the temperature was 60°C, borate had the highest complexing ability to d‐tagatose. When the temperature was lower than 50°C,

the effect of borate catalyzes l‐AI to d‐tagatose was closed to phosphate, which may be that the temperature inhibited the activity of l‐AI, and the d‐tagatose content was less; the borate complexation was not utilized (Figure 15).

#### 3.3.3 Borate catalyzes the optimal amount of enzyme added of l **‐AI**

**Figure 16:**
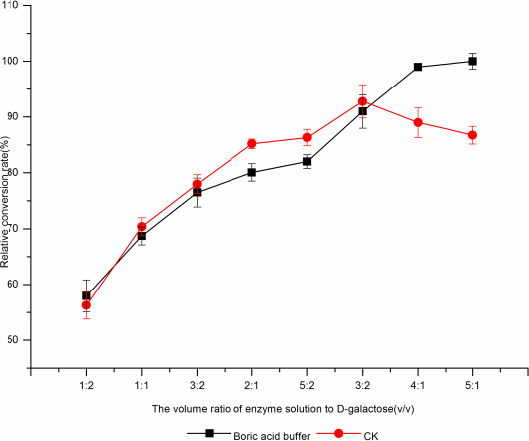
The effect of different enzyme amount and galactose volume ratio on borate‐catalyzedd‐tagatose

When the volume ratio of whole cell catalysts to d‐galactose was 5:1, the conversion rate of d‐tagatose was the highest, and as the volume ratio of enzyme to d‐galactose increased, the conversion rate of d‐tagatose was gradually increased, which may be the reason that the amount of d‐galactose was unchanged in the reaction system, the amount of whole cell catalysts was less, and only a small amount of enzyme protein was combined with the substrate, as the whole cell catalysts increased. d‐tagatose conversion was gradually increased. When the volume ratio of whole cell catalysts to d‐galactose was 5:1, the conversion rate of d‐tagatose was the highest (Fig. 16) (Lim et al., 2007; Xiaohui, 2012).

#### 3.3.4 The optimal addition amount of borate

**Figure 17:**
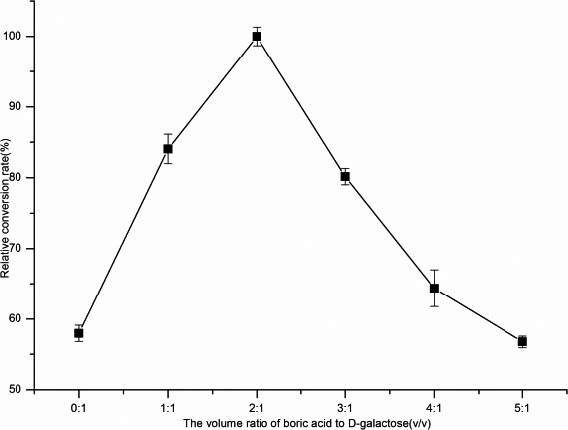
The effect of different borate additions on catalyticd‐tagatose

When the volume ratio of borate to d‐galactose was 2:1, the conversion rate of d‐tagatose was the highest, which was nearly 12% higher than the system without borate, and the yield of d‐tagatose was 58 g/L (Figure 17). Since then, the conversion rate of d‐tagatose had decreased with the increase of the amount of borate added, which may be the reason that the volume of borate added increased, and the concentration of d‐galactose gradually decreased, which was not conducived to l‐AI catalyzed the conversion of d‐galactose to d‐tagatose (Xiaohui, 2012).

## 4 CONCLUSION

In the study, *L. plantarum* WU14 was used as the d‐tagatose biotransformation fermentation starter, could determine the changes of each parameter in the fermentation process, and could establish three fermentation kinetic models to reflect the change rule of d‐tagatose biotransformation. The separation process of d‐tagatose could be optimized through decolorization, deproteinization, and desalination techniques, and the complexation ability of borate and d‐tagatose could be used to further improve the conversion rate of d‐tagatose, which would lay the foundation for the bioconversion d‐tagatose of industrial production.

## CONFLICT OF INTEREST

The authors do not have any conflict of interest.

## AUTHOR CONTRIBUTIONS

BoXu contributed to funding acquisition, project administration, and writing—review and editing. Zhijun Sun contributed to methodology, software, and writing—original draft. Tingting Miao contributed to conceptualization and validation. Aiguo Yin contributed to resources and supervision. Hulin Qiu contributed to data curation. Yunyi Xiao contributed to formal analysis. Ying Li contributed to investigation. Jinping Hai contributed to visualization.

## ETHICAL APPROVAL

This study does not involve any human or animal testing.

## INFORMED CONSENT

Written informed consent was obtained from all study participants.

## Data Availability

The data that support the findings of this study are openly available at http://doi.org/[figshare], Dataset title is “Optimization of Fermentation Conditions for Production of l‐Arabinose Isomerase of Lactobacillus plantarum WU14‐Raw data,” and the dataset link is https://doi.org/10.6084/m9.figshare.12986096.v1 (Sun et al., 2020).
